# Decisions to Withhold Diagnostic Investigations in Nursing Home Patients with a Clinical Suspicion of Venous Thromboembolism

**DOI:** 10.1371/journal.pone.0090395

**Published:** 2014-03-10

**Authors:** Henrike J. Schouten, Huiberdina L. Koek, Marije Kruisman-Ebbers, Geert-Jan Geersing, Ruud Oudega, Marijke C. Kars, Karel G. M. Moons, Johannes J. M. van Delden

**Affiliations:** 1 Julius Center for Health Sciences and Primary Care, University Medical Center Utrecht, Utrecht, the Netherlands; 2 Department of Geriatrics, University Medical Center Utrecht, Utrecht, the Netherlands; Wayne State University School of Medicine, United States of America

## Abstract

**Background:**

This study aimed to gather insights in physicians' considerations for decisions to either refer for- or to withhold additional diagnostic investigations in nursing home patients with a suspicion of venous thromboembolism.

**Methods:**

Our study was nested in an observational study on diagnostic strategies for suspected venous thromboembolism in nursing home patients. Patient characteristics, bleeding-complications and mortality were related to the decision to withhold investigations. For a better understanding of the physicians' decisions, 21 individual face-to-face in-depth interviews were performed and analysed using the grounded theory approach.

**Results:**

Referal for additional diagnostic investigations was forgone in 126/322 (39.1%) patients with an indication for diagnostic work-up. ‘Blind’ anticoagulant treatment was initiated in 95 (75.4%) of these patients. The 3month mortality rates were higher for patients in whom investigations were withheld than in the referred patients, irrespective of anticoagulant treatment (odds ratio 2.45; 95% confidence interval 1.40 to 4.29) but when adjusted for the probability of being referred (i.e. the propensity score), there was no relation of non-diagnosis decisions to mortality (odds ratio 1.75; 0.98 to 3.11). In their decisions to forgo diagnostic investigations, physicians incorporated the estimated relative impact of the potential disease; the potential net-benefits of diagnostic investigations and whether performing investigations agreed with established management goals in advance care planning.

**Conclusion:**

Referral for additional diagnostic investigations is withheld in almost 40% of Dutch nursing home patients with suspected venous thromboembolism and an indication for diagnostic work-up. We propose that, given the complexity of these decisions and the uncertainty regarding their indirect effects on patient outcome, more attention should be focused on the decision to either use or withhold additional diagnostic tests.

## Introduction

Both the annual incidence and the mortality rate of venous thromboembolism (VTE, deep vein thrombosis (DVT) or pulmonary embolism(PE)) rise considerably with increasing age [1], [2]. Diagnosing VTE is particularly challenging in older patients as symptoms and signs are nonspecific and might be camouflaged by co-morbidity in these patients [3]–[6]. Moreover, the specificity of D-dimer tests (e.g. the commonly used high sensitive ELISA-assays or latex agglutination assays) decreases with age to only 15% in patients aged 80 years and over [7], [8]. As imaging examination is indicated for those with an abnormal D-dimer test or a high probability of VTE obtained by application of a clinical decision rule, many older patients are being referred to a hospital for imaging examination (e.g. compression ultrasonography for DVT or CT pulmonary angiography for PE; procedures not typically available in primary care or in nursing homes). Nevertheless, many of these patients do not have VTE (typically 15 to 20% of older patients who undergo imaging examinations for clinically suspected venous thromboembolism are actually affected) [7], [9].

Prior work has shown that frail older patients are vulnerable to distress and complications resulting from transitions to hospital-care [10]–[12]. Gillick et al found that hospitalisation was associated with psychological and physiological symptoms (e.g. confusion, falling and incontinence) in 40% of hospitalized older patients (>70 years as compared to 9% in patient <70 years), irrespective of the medical diagnosis [13]. Yet, the burden and risks of hospital-attendance are of particular concern in these patients. Moreover, contrast enhanced computed tomography of the pulmonary arteries can cause nephropathy [14]. Though additional imaging examinations might prevent the sequelae of a missed diagnosis in a number of patients by directing appropriate treatment decisions, many will be exposed to the potential harms of referral for additional diagnostic work-up. Currently, there is growing concern that VTE might be overdiagnosed and thereby overtreated because of lower thresholds for application of increasingly sensitive imaging tests [15], [16]. Yet, little light has been shed on the actual burden and risk of the procedure of diagnostic investigations itself or to physicians' decisions to either refer for- or withhold diagnostic investigations (‘non-diagnosis decisions’) in older patients with suspected VTE. Therefore, this study aimed to explore physicians' considerations in such decisions [17], [18].

## Methods

A mixed-method study consisting of two parts was performed. In the first part, we quantitatively approached reasons for non-diagnosis decisions and compared the characteristics and patient-outcomes of the referred patients to those of the non-referred patients. Second, for a better understanding of the reasons underlying these decisions, we performed a qualitative study, applying the grounded theory approach and semi-structured in-depth interviews [19], [20].

### The quantitative approach

This study was nested in the Venous Thromboembolism in the Elderly-study (VT-elderly study) which aimed to quantify the accuracy of two diagnostic decision rules to diagnose or refute VTE in nursing home patients and community dwelling elderly patients across the Netherlands. The study had an observational and pragmatic design. Between October 2008 and April 2013, consecutive patients with a clinical suspicion of VTE were included by their physician (general practitioners for community dwelling patients, elderly care physicians for patients residing in nursing homes) [21]. Patients were not eligible for inclusion if they received anticoagulant treatment (vitamin K antagonists or oral direct thrombin- or factor Xa-inhibitors) at presentation or if they declined providing informed consent. Each patient's medical history, clinical characteristics, signs and symptoms, results on the diagnostic decision rule under study (the Wells score for patients primarily suspected of PE or the Oudega rule for patients primarily suspected of DVT) and on the D-dimer test-result were systematically recorded (Clearview Simplify D-dimer assay, Inverness Medical Princeton, NJ USA). Appendix S1 shows an overview of the characteristics of the Oudega-rule for DVT and of the Wells' rule for pulmonary embolism [22], [23]. Three months after inclusion it was verified whether the participant was still alive and if thromboembolic or bleeding-complications had occurred. Though referral for imaging examination (that is, compression ultrasonography of the entire proximal deep vein system in case of a suspicion of DVT, or CT-pulmonary angiography of VQ scanning when PE was suspected) was recommended for all patients with a high clinical suspicion of VTE, it was left to the physicians' discretion whether patients were indeed referred. This high clinical suspicion of VTE was based on either an abnormal D-dimer test or on a score >4 points on the Wells-rule for patients primarily suspected of PE; or on a score >3 on the Oudega-rule for patients primarily suspected of DVT [22], [23]. The referred patients with confirmed VTE were treated with coumarins and –until a stable INR in the therapeutic range was achieved- with a therapeutic dose of low molecular weight heparin. Patients in whom VTE had been refuted received no anticoagulant treatment. For the non-referred patients with a high clinical suspicion of VTE, it was left to the physicians' discretion whether patients received anticoagulant treatment. Physicians who decided to withhold referral for imaging examination in participants with a high risk of VTE were requested to identify appropriate reasons for this decision. For the current analysis, we included only patients residing in nursing homes with a high clinical suspicion of VTE. Within this group, we tested the differences between patients referred for additional diagnostic testing and non-referred patients, regarding patient characteristics and three-month bleeding rate and –mortality, according to received treatment. To assess to what extent the differences in the referred and non-referred groups contributed to their outcomes (i.e. potential confounding by indication) we calculated the probability of being referred for further diagnostic investigations based on the patients' characteristics (i.e. propensity score-estimation) and subsequently adjusted for this probability in a multivariable model. We used Statistical Package for Social Sciences (SPSS) version 20 for these analyses.

### Ethics statement

This study was judged as exempt from review by the local ethics review board of the University Medical Center Utrecht, the Netherlands (08-124/E) and conducted according to the Federation of Medical Scientific Societies' code of conduct for health research [24].

### The qualitative approach

#### Participants and data collection

Within the VT-elderly study, we qualitatively focussed on physicians' decisions to forgo referral for diagnostic investigations. By applying the “grounded theory” approach we set out to gain a higher level of understanding on the quality, that is the context wherein- and the perspective from which physicians decided to withhold further diagnostic investigations in nursing home residents with suspected venous thromboembolism. This understanding is “grounded” in a close and systematic analysis from in-depth interviews. The “grounded analysis” is based on three key principles: 1) simultaneous cycles of data collection and analysis (iterative analysis), 2) wherein emerging themes are refined and explored in the next interviews with participants who might have different perspectives (purposeful sampling), and 3) by comparison of issues of interest in the data with other examples for similarities and differences (constant comparison) [20], [25].

We purposefully sampled elderly care physicians who included one or more patients for whom it was decided to forgo referral for imaging examination despite a high risk of VTE. To diminish recall bias, only inclusions between January 2011 and May 2012 were selected, as the interviews were held between May and July 2012. Of 26 eligible elderly care physicians, 21 physicians (84%) participated, 4 physicians declined participation and one person was no longer employed as an elderly care physician. The five non-participating physicians (3 females, 4 from rural areas and one from an urban area) had all enrolled one patient for whom they withheld further investigations (2 patients primarily suspected of PE and 3 of DVT) and provided the following reasons for their decisions: ‘alternative diagnosis more likely’ and ‘advanced dementia’. These reasons and characteristic were comparable to those of the 21 participating physicians. The participating physicians were on average 52 years old and had an average of 20 years of experience as board certified elderly care physician; a medical specialty in the Netherlands in nursing home and primary care geriatric medicine [21]. None of the participants had affiliations with hospitals or with universities. The majority of the participants was female and most physicians provided care to patients with psychogeriatric disorders as well as to patients with somatic disabilities (table 1). The physicians underwent individual in-depth interviews, approximately 45 minutes in length at their workplaces, at a time chosen by the physicians. All physicians gave oral consent prior to the interview. To increase recall and to find a joint starting point, the interviewers (MK or HJS) introduced each interview with a résumé of the clinical situation of the patient for whom the decision to withhold additional diagnostic testing was made. Afterwards, the physicians were asked to describe the situation of the patient and to discuss their decision in detail. A topic list based on discussion and a systematic review of the research group was used at the end of each interview to ensure that all topics were discussed [17]. The interviews were conducted and analysed through constant comparison; after each interview the topic list was reviewed and modified according to the topics emerging from the interviews. After 13 interviews were performed, saturation was reached for the major concepts; this was confirmed with eight subsequent interviews. Consistency among the interviewers was encouraged by giving each other verbal feedback after each interview.

**Table 1:** Characteristics of the participants in the qualitative study.

**Response rate (%)**	21/26 (81)
**Participants in current study (total included, %)**	21 (100)
Age (median, range)	52 (37 to 61)
Work experience as elderly care physician (years, median, range)	20 (4 to 27)
Female (%)	15 (71.4)
**Patient population under physician's care** *	
Patients with psychogeriatric disorders (%)	20 (95.2)
Patients with somatic disorders (%)	17 (80.1)
Rehabilitation patients (%)	4 (19.0)
Palliative care patients (%)	3 (14.3)
Patients with psychiatric disorders or non-congenital brain injury(%)	2 (9.5)

* 17 physicians had more than one type of patients' populations.

#### Data analysis

Data collection alternated with data analysis. Interviews were audio-recorded, professionally transcribed verbatim, anonymized and checked for accuracy. Data was analysed according to the steps described in the QUAGOL [26]. Narrative reports were written after each interview and memos were formulated during the analytical phase to enhance a consistent analysis process. After reading and rereading the data, two researchers marked each meaningful text segment separately and developed preliminary codes based on the first six interviews (open coding, MK and HJS) [27]. The subsequent seven interviews were also separately coded by the two researchers. During joint meetings, they constantly compared their analysis to identify common themes and worked towards consensus in interpretation of the data (researcher triangulation) [20]. The subsequent eight interviews were similarly coded by one investigator (MK) and checked by a second investigator (HJS) (axial coding). A third investigator was consulted (HLK) to resolve discrepancies between the first two investigators. Afterwards, the interpretations of each code were specified and their appropriateness was monitored. Simultaneously constant comparisons within and across the preliminary categories were iteratively made to examine interrelationships between the categories that provided the basis for a theoretical framework. Interdisciplinary sessions were regularly held to review and appraise the emerging patterns (researcher triangulation, HLK, JJvD, MK and HJS), there was no substantial disagreement between the researchers during these sessions [20]. During all phases of the analyses, alternative explanations of the findings were proposed and discussed to ensure strictly inductive and data-driven formulation of concepts [26]. Data-analysis was supported by NVivo 10 software.

#### Rigour

After all interviews and analysis were performed, a focus group meeting took place in order to obtain peer review of the results. The participants of this focus group were 7 physicians (not being respondents in the interviews) employed in nursing homes within one organisation in Utrecht, the Netherlands. One investigator (HJS) presented the theoretical framework and invited the group to critically reflect on this concept in a reciprocal dialogue [20]. The presented model was acknowledged by the focus group at large; the meeting gave no cause to collect extra data.

## Results

### Quantitative approach

A total of 423 nursing home residents with clinically suspected VTE were enrolled in the VT-elderly study (294 patients primarily suspected of DVT and 129 of PE) of whom 322 patients had a high probability of VTE and/or an abnormal D-dimer test. Referral for additional diagnostic investigations was forgone in 126/322 (39.1%) patients. Anticoagulant treatment was initiated in 95 (75.4%) of these 126 patients in whom an objective diagnosis was lacking. The presence of co-morbidity and ‘a limited life-expectancy’ were most frequently indicated by physicians as reasons for their decision to withhold additional diagnostic imaging examination (respectively 73.8% and 50.0%; table 2).

**Table 2:** Reasons given by physicians (n = 84) to withhold additional investigations; indicating more than one reason was allowed.

Reason	Frequency of given reason (% of physician indicating the reason)
Co morbidity	62 (73.8)
Limited life-expectancy	42 (50.0)
Limited quality of life	30 (35.7)
Agreed palliative policy	27 (32.1)
Agreed symptomatic policy	22 (26.2)
Contra-indication anticoagulant treatment	6 (7.3)
Unusual in our nursing home	3 (6.0)

The non-referred patients were more often bedridden or chair-bound (respectively 68.5% versus 52.0%, p = <0.01), more often primarily suspected of PE instead of DVT (48.4% versus 20.1%, p<0.01) and had a higher score on the clinical decision rule compared to the referred patients (table 3). Moreover, the 3month mortality rates were higher in patients in whom investigations were withheld than in the referred patients, irrespective of anticoagulant treatment (31.0% versus 17.1%, odds ratio crude 2.15 (95% confidence interval 1.26 to 3.67) and odds ratio corrected for treatment 2.45 (1.40 to 4.29); table 4 and table 5). However, after adjustment for the probability of referral for additional diagnostic investigation (i.e. propensity scores) there was no significant difference in mortality between the non-referred and the referred patients (odds ratio 1.75 (0.98 to 3.11)). Moreover, there were no significant differences in bleeding rates between the referred and non-referred patients; no bleeding occurred in any patient who was not treated with anticoagulant treatment.

**Table 3:** Baseline characteristics, patients referred and not referred for additional diagnostic testing.

Patients with a high risk of VTE in whom imaging examination was indicated	Patients referred for investigations	Non-referred patients	p
n = 322	n = 199	n = 126	(*x_2_)*
**Demographic characteristics**			
Male	56 (28.1)	35 (27.8)	0.94
Age mean (SD)	82.3 (9.0)	82.3 (10.6)	0.45^a^
**Symptoms and signs**			
Acute onset of symptoms	138 (69.3)	84 (66.7)	0.61
Duration of symptoms in days, median (interquartile range)	2.0 (4.0)	3.0 (6.0)	0.10
Painful leg	91 (45.7)	38 (30.2)	<0.01
Swollen leg	158 (79.4)	67 (53.2)	<0.01
Erythema of leg	78 (39.2)	33 (26.2)	0.02
**Clinical probability of VTE**			
Physicians' estimation of the probability of VTE (Gestalt) in %, median (interquartile range)	65 (30)	70 (33)	0.62^b^
D-dimer abnormal	195 (98.0)	121 (96.0)	0.30
**Medical history and functionality**			
Previous DVT	22 (11.1)	10 (7.9)	0.36
Previous pulmonary embolism	14 (7.0)	8 (6.3)	0.81
Active malignancy	26 (13.1)	17 (13.5)	0.91
Bedridden or chairbound (i.e. unable to walk)	103 (52.0)	85 (68.5)	<0.01
**Outcomes within 3 months**			
Anticoagulant treatment initiated without confirmation of the diagnosis	-	95 (75.4)	-
VTE confirmed	118 (59.3)	-	-
Clinical significant bleeding	6 (3.0)	9 (7.1)	0.08^e^
3months mortality	34 (17.1)	39 (31.0)	<0.01^e^
**Patients primarily suspected of DVT**	**n = 159**	**n = 65**	
Difference in calf circumference in cm, mean (SD)^c^	3.8 (2.0)	3.4 (2.2)	0.93
Oudega score for DVT (clinical variables only), mean (SD)^c^	2.6 (1.5)	2.2 (1.7)	0.24^a^
DVT confirmed	22 (55.0)	-	
3monts mortality	25 (15.7)	14 (21.5)	0.30
**Patients primarily suspected of PE**	**n = 40**	**n = 61**	
Cough^d^	8 (20.0)	11 (18.0)	0.81
Pain at inspiration^d^	17 (42.5)	17 (27.9)	0.13
Dyspnoea^d^	31 (77.5)	52 (85.4)	0.80
Tachycardia (>100 per minute)^d^	13 (32.5)	28 (45.9)	0.18
Total score on the Wells rule for pulmonary embolism, mean (SD)^d^	4.5 (1.9)	3.6 (2.2)	0.28^a^
Pulmonary embolism most likely diagnosis^d^	30 (75.0)	31 (50.8)	0.02
PE confirmed	46 (70.8)	-	-
3months mortality	9 (22.5)	25 (41.0)	0.06

a Independent samples T-test;

b Mann- Witney U-test;

c Data only available for patients primarily suspected of DVT;

d Data only available for patients primarily suspected of pulmonary embolism;

e provided p-values over the total groups of patients. The p-values within the strata ‘primarily suspected of PE’ or ‘primarily suspected of DVT’ were >0.05.

**Table 4:** Multivariable association with decisions to withhold additional diagnostic testing; stepwise backward selection of variables.

Variable	Odds ratio (95% confidence interval) for physicians' decision to withhold additional investigation
Total score on clinical decision rule	0.86 (0.75 to 0.99)
Chair bound or bedridden (reference = able to walk)	1.96 (1.18 to 3.25)
Initial suspicion PE (reference = primary suspicion of DVT)	0.21 (0.12 to 0.36)

**Table 5:** The association of decision to withhold diagnostic testing with patient outcomes within 3 months.

	3 month mortality	3 month bleeding rate (any clinically significant bleeding)
Non diagnosis decisions (crude)	2.15 (1.26 to 3.67)	2.60 (0.90 to 7.48)
Non diagnosis decisions (treatment added)	2.45 (1.40 to 4.29)	2.24 (0.76 to 6.60)
Non diagnosis decisions (Propensity score* added as continuous variable)	1.75 (0.98 to 3.11)	2.78 (0.90 to 8.60)
Non diagnosis decisions (Propensity score* and anticoagulant treatment added)	1.99 (1.09 to 3.62)	2.38 (0.75 to 7.54)

Odds ratios (95% confidence interval).

* Propensity score for the probability of referral for further diagnostic investigations based on the following variables: gender, age, mobility, primary suspicion DVT or PE, duration of symptoms, acute onset, painful leg, swollen leg, previous DVT, previous PE, decubitus, antiplatelet use, estimated probability of VTE by physician, total score on decision rule. There was a moderately to good balances for all variables within the propensity –scores.

### Qualitative approach: motivations for non-diagnosis decisions

Further analyses were restricted to the qualitative analysis of the in-depth interviews. In the physicians' reasoning, three key-themes were identified. These key-themes were translated to three key-questions describing the most important reasons in the physicians' consideration of the proportionality (that is the harm-benefit ratio) of the referral for additional diagnostic interventions (table 6): 1) What is the relative impact of the potential disease? 2) Does performing additional diagnostic investigations agree with advance care planning? 3) And, do potential benefits of additional diagnostic investigations outweigh burden and risks for the patient? Furthermore, physicians named several non-patient related factors that influenced their decisions; we called these factors ‘modulating factors’ (table 7).

**Table 6:** The main categories in the physicians' considerations.

Key question	Considerations inclining the physician to refer the patient for additional imaging examination	Considerations inclining the physician to withhold referral additional imaging examination	Citations illustrating the consideration
**What is the relative impact of the potential disease?**	**Potential disease;** risks/threats of the disease for the patient's prognosis; mortality risk; severity and burden of current symptoms; expected burden of potential complications of disease	**Chronic condition of the patient;** low quality of life; high age; worse prognosis/short life expectancy; cognitive decline; (irreversible) chronic burden of disease	*I1: “The spinal cord lesion and the paraplegia determine the rest of her life, irrespective of how long that may be. It is of course already an old lady. And in my experience, it does not make sense to mess up things for a particular detail, such as a complication of thrombosis. This might seem strange, but with all these major miseries, it is just a detail.”*
**Does performing investigations agree with the goals for medical interventions as established in advance care planning?**			*I13: “Consider a patient (…) who is in a preterminal phase. In such a case we focus on the prognosis and life expectancy. Which complications may occur when we refrain from actions? And how does that affect the quality of life?”. I12: “So, here is actually a woman of whom we disrespectfully say “this is someone who has forgotten to die.” Perhaps, this may sound bad, but she really is not happy. So, we secretly hoped for that this would be her time to finally die”*
**Do the potential benefits of the investigation outweigh its burden and risks for the patient?**	**Potential benefit of diagnostic investigation**	**The burden and risks of investigation**	*I21: “In a nursing home it is not obvious to exhaust all possibilities and resources. Almost all things you do is a consideration of the expectancy and the burden of something, and also the expected course afterwards.”*
	***Alteration in management;*** The likelihood that further investigations will alter intended management	***Physical burden of investigation;*** duration of hospital visit; transport to hospital; physical complication risk	*I16: “Sure, you act in good conscience, also in this case. Yeah, you never know for certain, but the clinical picture gives me the impression that there is a high probability the diagnosis is correct.” I14:“I consider this as a great burden: in the ambulance, lying there for hours, bearing several examinations, family that has to accompany…. and then returning several hours later reporting; ‘the examination has failed’.”*
	***Proportionality of the burden of treatment;*** establishing the diagnosis makes the burden of treatment more proportional.	***Burden of treatment;*** Risk of (bleeding) complications; Burden of drug administration and monitoring	*I14: “If one frequently falls and there are signs of PE, therefore you should treat, but you also know that one falls and could even get an intracranial haemorrhage, then –with a person who is fine- the priority of the diagnosis takes over the argument.”*
	***Added value for the patient's quality of life;*** through assessment of prognosis or through guidance and care for the patient	***Mental, psychological and emotional burden and coping***; Not understanding what is going on; unable to lie still; offering resistance; risk of mental complications(e.g. delirium)	*I12: “What counts as well is that, in many of my years of experience, I have seen so much misery: people going to the hospital and either dying there, tremendously delirious, tied up to the bed, or returning in a condition that makes you say: “Oh my, I wish we had never started this.”*
			*I9: “Well, in her case it also played a role that the confirmation of the diagnosis did not outweigh the increasing risk of delirium by doing these kind of things ”*

**Table 7:** Modulating factors.

**Physician related factors**	
Experience	Duration of work as physician in elderly care
	Feedback on own acting
	Medical training
Standards and values	Not wanting to do medically pointless interventions
	Though aware of it, costs of medical interventions are no deciding factor
	Starting or continuing interventions is considered easier than stopping or withdrawing interventions
	Physician takes (responsibility for) decision and tries to get the patient ('s family) to go along
	Aim to prevent a conflict with patient('s family)
Professional standards	In general being reserved to refer to a hospital
	Little available diagnostic technology in the nursing home lead to more often withholding it
	Curiousness or ‘wanting to know’ of less importance
	Holistic patient approach
	Pursuit of quality of life and comfort
	Being aware of the finiteness of life
Fear for losing direction when referring	Risk for more diagnostic interventions than requested
Diagnostic uncertainty	
**Patient('s family) related factors**	
Patient's wish	Negative experience with previous hospital admissions
Derived patient's wish	Wanting to reduce the duration of the patient's suffering
	Previous statements of the patient which support restraint management
Family	Negative experience with patient's previous hospital admissions
	Unable to take leave of the patient
	Unable to handle uncertainty
	Having a feeling of guilt
	Considered burden of the patient for the informal caregiver
	Composition of the family and family bonds
Religion/culture	Religious patients tend to wish to continue medical interventions to the very end
**Circumstances**	Distance to hospital
	Availability of diagnostic interventions
	Availability of someone to accompany the patient
	Time of the day/week
	Workload
	Inconvenience to arrange referral
**Other factors**	Conceived burden of the referral for the caregivers in the hospital
	Characteristics of the nursing home hardly influences the decision making
	Not knowing the patient inclines the physician to referral

#### Key question 1: What is the relative impact of the potential disease?

The impact of the potential VTE-event was estimated (that is, a combination of the severity of symptoms and estimated prognosis) and was considered in the perspective of the patient's chronic condition. For some patients, the impact of the potential VTE-event was overshadowed by their chronic condition; physicians expected that the potential VTE-event would not significantly alter their quality of life or life-expectancy as this was largely determined by their chronic condition. For example, a physician of a patient with paraplegia due to a spinal cord lesion considered the suspected DVT as ‘just a detail’ for his patient (table 6) which was the main reason to withhold further diagnostic work-up for this patient. However, the presence of more severe symptoms (e.g. severe discomfort due to suspected PE) or severe complication risks inclined physicians to perform additional diagnostic tests.

#### Key question 2: Does performing of additional diagnostic investigations agree with advance care planning?

Physicians stated that it is common practice to discuss advance care planning with every resident at their admission in Dutch nursing homes. Advance care planning implies a decision concerning the outline of the goals and boundaries for medical interventions based on regularly held discussions with the patient or his/her legal representative. Next to the patient's chronic condition and estimated prognosis, the patients' attitude and his/her (negative) experiences with previous hospital admissions commonly played a role in the goals of medical interventions. Physicians experienced these predefined advance care planning as guiding principles for their medical decisions, next to the wish of the patient and his or her family at the time of the clinical suspicion of VTE. Though referral to a hospital was generally considered inappropriate within a “palliative-” or “symptomatic goal” (i.e. medical treatment aimed at optimal well-being and an acceptable quality of life rather than on cure or extension of life) [28], it was generally believed that anticoagulant treatment would relieve the complaints of the patient and therefore it was considered as an appropriate intervention for patients with such an in-advance planned “palliative-” or “symptomatic goal”. However, one physician consciously decided- in consultation with the patients' representatives- to withhold anticoagulant treatment for a patient and hoped that the possible PE would be an opportunity to let the patient pass away (table 6); for this patient, the pre-determined goal of medical care was to optimize well-being rather than on cure or extension of life.

#### Key question 3: Do the potential benefits of the investigation outweigh its burden and risks?

In the light of relative impact of the potential disease, the potential net-benefits of investigations were estimated. Physicians stated that the performance of investigations driven by curiosity or ‘just to know the diagnosis’ did not fit in their professional standards (table 7). The pursuit of a diagnosis was considered of limited value if this would not lead to an alteration in management.

Several physicians seemed to strongly rely on their diagnostic reasoning: they estimated the probability of VTE (based on clinical signs and symptoms, D-dimer testing) as very high (“there was no alternative explanation for these symptoms”) and subsequently immediately started anticoagulant treatment. In their opinion, anticoagulant treatment would have been initiated anyhow, so they considered imaging examination of limited value. In contrast, several physicians would only start anticoagulant treatment if the diagnosis of VTE would be confirmed by imaging examination and considered the complication risks of treatment unacceptable if the diagnosis would not be established (table 6). Others withheld treatment in particular patients as they judged the disadvantages of the treatment – either due to complication risk or burden of the administration and monitoring- of overriding importance.

Physicians felt that the transport to a hospital and undergoing additional investigations would bring on physical and mental burden to their patients. It was felt that hospital care was not sufficiently set up for frail older people. Fear of disturbing the patient's mental equilibrium was another reason cited by physicians to not seek additional diagnostic tests. Physicians considered that a hospital admission would strain their coping and that it could even be detrimental due to complications. Particularly for patients with cognitive decline or psychiatric diseases, referral was considered burdensome. For some patients it was felt that it would even be impossible to perform imaging examinations, as they would get restless because they would not understand what was going on, or that they would offer resistance.

### Modulating factors

Next to the considerations of the proportionality of investigations for a particular patient, we detected several factors that affected the physicians' decisions more in general (listed in table 7). As a result of their decision to withhold diagnostic investigations physicians felt that they had to accept more uncertainty in their treatment decisions. Physicians with more work experience tended to be less concerned by this uncertainty and placed greater emphasis on their clinical judgement. Moreover, their estimations of the relative benefits of investigations for the patients tended to be less positive. Some physicians expressed the fear that their lead role in decision-making would be lost if patients were referred to the hospital. Others experienced resistance from hospital workers if they intended to hand over a patient to hospital care. In addition, various practical considerations could also persuade the physician to forgo referral; for example the inconvenience of arranging a referral or the absence of someone to accompany the patient.

## Discussion

This study explored physicians' decisions to withhold diagnostic investigations in elderly patients, both in a quantitative and qualitative manner. We found that almost four out of ten nursing home patients with a high risk of VTE were not referred for additional diagnostic investigations. Generally, elderly care-physicians considered referral for additional diagnostic testing as a great burden for their frail older patients and aimed to reserve referrals for problematic cases. This was in line with previous studies pointing out the risks and burden of hospital transfers in frail older patients [11], [12], [29]. Hospital-transitions among nursing home residents are associated with in increased risk of functional decline, development of decubitus ulcers, tube feeding insertion (adjusted odds ratios up to 2) and a 20% risk of adverse drug events due to prescription errors [10], [13], [30], [31]. Compared to the patients who were referred for additional diagnostic investigations, the non-referred patients had a higher crude 3month mortality rate; almost one out of three of these patients died within three months. Due to the non-randomized design of the study we cannot firmly interpret these findings. Though it is possible that the lack of an adequate diagnosis and subsequently under- or overtreatment partly contributed to the higher mortality-rates in the non-referred patients [32], it is much more likely that the worse outcomes of the non-referred patients can be explained by a worse prognosis of these patients non-referred beforehand; compared to the referred patients, the non-referred patients were more often primarily suspected of PE (instead of DVT) and more often severely impaired in their mobility. Moreover, though univariable analysis revealed a higher 3month mortality-rate for the non-referred patients, there was no longer an association between non-diagnosis decisions and mortality when the probability of being referred (i.e. the propensity score) was added to the multivariable model. Yet, the differences in 3month mortality largely derives from differences in patient characteristics rather than by the effect of the decision to withhold diagnostic investigations and subsequently guided therapy. Therefore, our results raise the important management question whether the potential (but unknown) benefit of definite diagnosis versus empirical treatment outweighs the known harms of hospital transfer in nursing home residents with a clinical suspicion of venous thromboembolism in whom an objective diagnosis is lacking.

Strikingly, anticoagulant treatment was initiated in most (75%) of the patients for whom was decided to withhold investigations. Though there was a general belief among physicians that anticoagulation treatment would relieve the complaints of their patients, there appeared to be a large variation in the physicians' notions on the risks and benefits of anticoagulant treatment in older patients and in the subsequent effects of these notions on their decisions. Several physicians considered the complication risks of anticoagulant treatment insignificant and were inclined to initiate treatment without confirmation of the diagnosis, whilst others considered the bleeding risk as substantial and were only willing to initiate treatment if the diagnosis was objectively confirmed, whereas others decided to withhold further diagnostic testing as they intended to withhold anticoagulant treatment irrespective of the diagnosis. Previous studies showed that in older patients with multi-morbidities, anticoagulation treatment is associated with a more than twofold increased bleeding risk [33]–[35]. However, despite this risk, anticoagulant treatment is highly effective in prevention of (fatal) recurrences of VTE (absolute risk reduction of 52.6% of fatal and non-fatal recurrences), and therefore, even high age, multiple comorbidities and/or cognitive impairment are not necessarily contra-indications for anticoagulant treatment [33], [36], [37].

The strengths of our study derive from the combined quantitative and qualitative methods to gain understanding of the physicians' diagnostic decision making and the context of- and important reasons in this diagnostic decision making. Furthermore, there was good concordance in the analysis of the researchers who separately and subsequently jointly reviewed transcripts. Also, validation of the results by means of the focus group meeting did not show serious disagreement with the analysis.

Yet, for full appreciation of our results, some aspects of our study warrant comment. First, our study was a post-hoc analysis on data of a prospective study which aimed to validate clinical decision rules in combined with normal D-dimer testing to rule out VTE in older nursing home patients. Consequently, not all variables identified in our qualitative study to potentially correlate with the physicians' decision to withhold additional diagnostic investigations were systematically collected in the quantitative study. Specifically, we did not determine a frailty index score, the presence of ‘do not resuscitate’ orders, or the presence of either cognitive or renal function impairment. Moreover, our study was not primarily powered to detect differences between referred and non-referred patients. A larger sample size would possibly have resulted in more significant differences between these two groups.

Second, the single-country of the study might hamper generalization of our findings to other countries, as the organization and healthcare ethics in the Dutch nursing home care may be different from other countries [21], [38]. Medical care for nursing home residents in the Netherlands is delivered by so called ‘elderly care physicians’; a medical specialty in the Netherlands in nursing home geriatric medicine. These physicians have completed a medical specialisation training of three years and –in general- exclusively deliver care to geriatric nursing home patients (i.e. not in hospital settings). In a qualitative study comparing decisions of Dutch and American physicians (from North Carolina) to treat or withhold treatment in nursing home residents with pneumonia, Helton and colleagues found that American physicians were more deferential to family preferences and were inclined to treat more aggressively, even in cases when they considered families' wishes for care as inappropriate [38]. Therefore, more studies -particularly in other settings and countries- are needed to further explore physicians' diagnostic reasoning and also to quantify the impact of additional diagnostic testing on patients' quality of life in clinically relevant subgroups.

Third, the semi structured face-to-face interview method offered a context for the physicians to speak honestly about difficult clinical situations and their considerations in their decision-making and the interviewers made every effort to stimulate the physicians' frankness. Nevertheless, the possibility of socially acceptable answers by the participants could not fully be excluded.

Last, though the pragmatic and observational study-design of the VT-elderly study did not force physicians to refer patients with high scores on the clinical decision rule, it is possible that physicians were less prone to include the frailest patients or patients in whom they deviated from the rule in the VT-elderly study (gatekeeping) [39]. This might have introduced selection bias which might have led to an underestimation of the frequency of non-diagnosis decisions for patients residing in Dutch nursing homes. Nevertheless, we do not expect that this hampered the completeness in the variety of our presented categories in the qualitative analysis of non-diagnosis decisions [20], [25].

In conclusion, our results suggest that elderly care physicians are frequently faced with the difficult task to decide whether referral for additional diagnostic investigations is of benefit to their individual patient with suspected VTE. For almost four out of ten nursing home patients with a high clinical suspicion of VTE, additional diagnostic investigations were withheld. ‘Blind’ anticoagulant treatment was initiated in three out of four of the non-referred patients. The 3month mortality rates were higher for patients in whom investigations were withheld than in the referred patients, irrespective of anticoagulant treatment. However, when adjusted for the propensity score, there was no relation of non-diagnosis decisions to mortality. We unravelled the physicians' complex decisions to forgo additional diagnostic investigations. Our analysis revealed that the physicians' decision to forgo additional diagnostic investigation was a complex one that appeared to be primarily based on their judgment of the benefit balanced against potential harms likely to come from such testing. Given the complexity of these decisions, more attention for this formerly undiscussed topic is needed. This may open debate among physicians and contribute to well-considered decision making.

## Supporting Information

Appendix S1 **Clinical decision strategies under study.**
(DOCX)
