# Supplementary material for: Decisions to Withhold Diagnostic Investigations in Nursing Home Patients with a Clinical Suspicion of Venous Thromboembolism
Source: PLoS One. 2014 Mar 10;9(3):e90395. doi: 10.1371/journal.pone.0090395 (PMC3948630; doi:10.1371/journal.pone.0090395)
Supplement: Appendix S1 — Clinical decision strategies under study. (DOCX) [file pone.0090395.s001.docx]

**Appendix S1- Clinical decision strategies under study**

| **Variables** | **Points** |
| --- | --- |
| **For DVT (Oudega strategy) [22]** |  |
| Male gender | 1 |
| Use of oestrogens (oral contraceptive use or hormonal replacement therapy) | 1 |
| Presence of malignancy | 1 |
| Recent surgery | 1 |
| Absence of leg trauma | 1 |
| Vein distension | 1 |
| Calf difference ≥ 3 cm | 2 |
| D-dimer abnormal | 6 |
| **For PE (Wells’ strategy) [9]** |  |
| Clinical signs and symptoms of deep vein thrombosis | 3.0 |
| Pulmonary embolism more likely than alternative diagnosis | 3.0 |
| Heart rate >100 beats/min | 1.5 |
| Immobilisation (>3 days) or surgery in previous four weeks | 1.5 |
| Previous pulmonary embolism or deep vein thrombosis | 1.5 |
| Haemoptysis | 1.0 |
| Malignancy (receiving treatment, treated in past six months, or palliative) | 1.0 |
